# Comparative Metabolic Analysis Reveals a Metabolic Switch in Mature, Hydrated, and Germinated Pollen in *Arabidopsis thaliana*

**DOI:** 10.3389/fpls.2022.836665

**Published:** 2022-05-18

**Authors:** Jiang Wang, Shrikaar Kambhampati, Doug K. Allen, Li-Qing Chen

**Affiliations:** ^1^Department of Plant Biology, University of Illinois at Urbana-Champaign, Urbana, IL, United States; ^2^Carl R. Woese Institute for Genomic Biology, University of Illinois at Urbana-Champaign, Urbana, IL, United States; ^3^Donald Danforth Plant Science Center, St. Louis, MO, United States; ^4^United States Department of Agriculture, Agricultural Research Service, St. Louis, MO, United States

**Keywords:** untargeted metabolomics, *in vitro* pollen germination, hydrated pollen, germinated pollen, metabolites, mature pollen, starch and sucrose metabolism

## Abstract

Pollen germination is an essential process for pollen tube growth, pollination, and therefore seed production in flowering plants, and it requires energy either from remobilization of stored carbon sources, such as lipids and starches, or from secreted exudates from the stigma. Transcriptome analysis from *in vitro* pollen germination previously showed that 14 GO terms, including metabolism and energy, were overrepresented in *Arabidopsis*. However, little is understood about global changes in carbohydrate and energy-related metabolites during the transition from mature pollen grain to hydrated pollen, a prerequisite to pollen germination, in most plants, including *Arabidopsis*. In this study, we investigated differential metabolic pathway enrichment among mature, hydrated, and germinated pollen using an untargeted metabolomic approach. Integration of publicly available transcriptome data with metabolomic data generated as a part of this study revealed starch and sucrose metabolism increased significantly during pollen hydration and germination. We analyzed in detail alterations in central metabolism, focusing on soluble carbohydrates, non-esterified fatty acids, glycerophospholipids, and glycerolipids. We found that several metabolites, including palmitic acid, oleic acid, linolenic acid, quercetin, luteolin/kaempferol, and γ-aminobutyric acid (GABA), were elevated in hydrated pollen, suggesting a potential role in activating pollen tube emergence. The metabolite levels of mature, hydrated, and germinated pollen, presented in this work provide insights on the molecular basis of pollen germination.

## Introduction

Successful pollination in flowering plants is essential to fertilization and seed formation and is a key determinant of seed yield (Johnson et al., 2019). Mature pollen in most plant species is metabolically dormant before anthesis with approximately 15–35% water content (Shi and Yang, 2010). Pollen viability correlates with the degree of dehydration and the composition of carbohydrate and lipid reserves (Shi and Yang, 2010). Upon interacting with stigma, compatible pollen grains will undergo a rapid rehydration process that is a prerequisite to germination (Moon and Jung, 2020). It takes less than 5 min for pollen to germinate in many monocot species (Heslop-Harrison, 1979; Chen et al., 2008). By contrast, dicot pollen can take an hour to hydrate before germination (Rotsch et al., 2017). Defects in pollen hydration may result in precocious germination in anthers (Johnson and McCormick, 2001) and cause sterility (Fiebig et al., 2000). After hydration, metabolism initiates pollen tube growth from the aperture, which is controlled by multiple cellular and molecular processes (Kim et al., 2019). The rapid growth of pollen tubes is an energy-demanding process that requires mobilization of storage reserves in pollen grains (Goetz et al., 2017) and support from stigma exudates (Goldman et al., 1994; Wolters-Arts et al., 1998) coordinated by a complicated change of metabolic dynamics, protein synthesis, cell signaling, cell-wall remodification, and new cell component biosynthesis (Shi and Yang, 2010; Johnson et al., 2019; Kim et al., 2019; Hafidh and Honys, 2021).

Studies performed with rice and *Arabidopsis* [see review in Moon and Jung (2020) and references therein] analyzed the complexity of biochemical mechanisms initiated during pollen germination and pollen tube growth. In contrast to the wealth of available transcriptome and proteome data, global metabolomic dynamics have not been well-characterized during pollen germination. In particular, knowledge of metabolomic changes during pollen hydration is nearly absent from the literature and metabolite reports on pollen are limited to a small number of specific compounds. Secondary metabolites have been examined in tomato plants during pollen development under heat stress (Paupiere et al., 2017) and sucrose and starch catabolism has been measured in different fractions in the lily (Castro and Clément, 2007). Comprehensive metabolomic analysis during pollen germination has been performed in lilies and tobacco, two bicellular pollen species (Obermeyer et al., 2013; Rotsch et al., 2017), and the Chinese fir, a gymnosperm (Fragallah et al., 2018), but no such studies have been reported with the model plant *Arabidopsis*, a tricellular pollen species, despite the extensive transcriptomic resources available for comparative analysis.

In this study, we examined changes in *Arabidopsis* pollen by surveying metabolite profiles among mature, hydrated, and germinated pollen with an untargeted metabolomic approach. Monosaccharide, polysaccharide, sugar phosphate, lipid, and fatty acid levels changed during the process. In addition, integrated analysis of the metabolomic and publicly available transcriptomic data revealed that sucrose and starch metabolism were significantly elevated in pollen hydration and germination. Several genes that encode transporters and enzymes for phosphorylated sugars as well as lipid synthesis enzymes were differentially expressed in accordance with the changes of their metabolic intermediates, suggesting some concordance with transcript data. The potential roles of several metabolites that were over-represented in hydrated pollen required for the metabolic activation of pollen tube emergence were discussed with reference to transcriptomic data.

## Materials and Methods

### Plant Growth and Pollen Collection

The *Arabidopsis* Col-0 plants were grown under controlled temperature (22°C) with a 16-h light (100–150 μmol m^–2^ s^–1^)/8-h dark photoperiod. The pollen harvesting method, *in vitro* germination medium, and sampling stage for hydrated pollen (45 min after germination) and germinated pollen (4 h after germination) for metabolomic analyses were identical to methods published based on transcriptome analysis of *Arabidopsis* pollen germination (Wang et al., 2008), to enable comparisons. For each repeat, mature pollen grains from the fully opened flowers were collected from more than 1,000 plants using a vacuum cleaner method (Johnson-Brousseau and McCormick, 2004) at around 5 h into the light period.

### Culture of *Arabidopsis* Pollen *in vitro*

For mature pollen samples, collected pollen grains were resuspended in 2 ml of ice-cold Pollen Isolation Buffer [PIB, composed of 100 mM NaPO_4_, pH 7.5, 1 mM EDTA, and 0.1% (v/v) Triton X-100] right after collection followed by centrifuging at 15,000 g for 1 min (4°C). For hydrated pollen and germinated pollen samples, in brief, pollen pellets were washed with 1 ml of liquid Pollen Germination Medium [PGM, composed of 15% (w/v) sucrose, 1.5 mM boric acid, 0.8 mM MgSO_4_, 1 mM KCl, 5 mM MES, 0.05% (w/v) lactalbumin hydrolysate, 10 μM myo-inositol, 5 mM CaCl_2_] before they were resuspended in 30 μl of liquid PGM and subsequently cultured in Petri dishes (35 mm in diameter). A 70 μm mesh was used to cover the pollen droplet to create a thin layer for optimal germination for each Petri dish. The Petri dishes were covered and placed in the dark for 45 min or 4 h and collected as hydrated pollen or germinated pollen, respectively. All pollen samples were washed by 1 ml ice-cold ddH_2_O three times before being stored in a -80°C freezer.

### Starch Staining of *in vitro* Germinated Pollen

Mature, hydrated, and germinated pollen were prepared as aforementioned. Pollen samples were stained using 100 μl iodine solution [composed of 4% (w/v) potassium iodide and 1.27% (w/v) iodine] kept in the dark for 10 min and washed twice using 1 ml ddH_2_O before imaged with a compound microscope (Nikon, NY, United States).

### Total Metabolite Extraction

Total metabolites from pollen were extracted using a phase separation method previously described (Kambhampati et al., 2021) with slight modifications. Briefly, 10–30 mg (fresh weight) pollen tissue, collected in Eppendorf tubes, was extracted using 700 μL of chilled 7:3 (v/v) methanol: chloroform spiked with 50 μM each of 1.4-piperazinediethanesulfonic acid (PIPES), ribitol, and norvaline as internal standards. After two metal beads were also added to the samples, they were homogenized using a Tissue-Lyser for 5 min at 30 Hz. The samples were incubated on a rotary shaker at 4°C for 2 h after which 300 μL ddH_2_O was added. The samples were then centrifuged at 14,000 rpm for 10 min to achieve phase separation and the upper aqueous phase, as well as the lower organic phase, were collected separately. The aqueous phases containing polar and non-polar metabolites were split into two equal parts and dried using a speed vacuum centrifuge (Labconco, Kansas City, MO, United States). The two dried parts were re-suspended in 50 μL 80% (v/v) methanol, and 30% (v/v) methanol for metabolomics analyses using hydrophilic interaction chromatography (HILIC) and reverse phase chromatography, respectively. The organic phase was also dried using a speed vacuum centrifuge and re-suspended in 50 μL of 49:49:2 (v/v/v) mixture of acetonitrile:methanol:chloroform. All samples were filtered using a 0.8 μM PES membrane centrifuge filter (Sartorius, Goettingen, Germany) and transferred to a glass vial for injection into an LC-MS/MS system.

### Liquid Chromatography-Tandem Mass Spectrometry

Three different chromatographic methods, including a HILIC, a reverse phased C18 and a reverse phased C8 columns, were used to attain a wide coverage of compound groups. The aqueous fraction of the extraction, which is expected to contain core and specialized metabolic intermediates, was used for HILIC and C18 chromatography, while the organic fraction was used for C8 based chromatography for the separation of lipids. An Eksigent Ekspert microLC 200-chromatography system (Eksigent Technologies, Redwood City, CA, United States) and a CTC Analytics Leap HTS PAL liquid handler hooked to a benchtop Q-Exactive Orbitrap MS (Thermo Scientific, Waltham, MA, United States) were used for all untargeted LC-MS analysis. HILIC separation was achieved using a custom made zic-pHILIC (100 × 0.5 × 3 μm) column obtained from Higgins Analytical Inc. (Mountain View, CA, United States) with the mobile phases, 10 mM ammonium bicarbonate in ddH_2_O (solvent A) and 10 mM ammonium bicarbonate in 95:5 (v/v) Acetonitrile: ddH_2_O (solvent B) and a flow rate of 15 μL/min. The following gradient was used for HILIC; 0-2 min at 100% B, 3 min at 85% B, 16 min at 50% B, 17 min at 30% B, 18 min at 30% B, 20 min back to 100% B and equilibration up to 30 min. Reverse-phase chromatography was performed using a Targa C18 (100 × 0.3 × 5 μm) column with the mobile phases, 0.1% formic acid in ddH_2_O (Solvent A) and 0.1% formic acid in Acetonitrile (Solvent B), and a flow rate of 15 μL/min. The gradient conditions used for the C18 method are as follows; 0-3 min at 2% B, 13 min at 100% B, 16 min at 100% B, 19 min at 2% B, and equilibration up to 30 min. For lipidomics, a custom-made C8 column (100 × 0.5 × 1.7 μm) from Higgins Analytical Inc. (Mountain view, CA, United States) was used with the mobile phases 1% 1 M ammonium acetate, 0.1% acetic acid in ddH_2_O (solvent A) and 1% 1 M ammonium acetate, 0.1% acetic acid in 7:3 (v/v) acetonitrile: isopropanol (solvent B), and a flow rate of 40 μL/min. The following gradient, 0–1 min at 55% B, 3 min at 75% B, 8 min at 89% B, 10 min at 99% B, 11 min at 99% B and 12 min at 55% B followed by equilibration up to 18 min, was used, which was modified from Hummel et al. (2011) to adopt to microflow.

Data for untargeted metabolomics using all three chromatographic methods were acquired for mass ranges of 70–1,000 m/z by full MS at 70,000 resolution in both positive and negative ionization modes. The automatic gain control (AGC) and maximum injection time (IT) were 5 × 10^5^ and 100 ms, respectively. The heated electrospray ionization (HESI) source was operated with sheath gas, 15 arbitrary units; auxiliary gas, 5 arbitrary units; capillary temperature, 250°C; auxiliary gas heater temperature, 50°C; and S-lens RF level, 50. The spray voltage was 4.2 and 3.9 kV in positive and negative modes, respectively. One sample in each group was also used for the top 12 data-dependent acquisition experiments in both ionization modes to generate MS/MS datasets for compound identification. These experiments involved a full MS scan at 70,000 resolution, AGC target 5 × 10^5^, maximum IT 100 ms and MS/MS scans at 17,500 resolution, AGC target 5 × 10^4^, maximum IT 50, 2.0 m/z isolation window, stepped collision energy of 15, 25, and 35 eV, intensity threshold 1 × 10^4^ and 15-s dynamic exclusion.

### Metabolic Data Analysis and Integration of Available Transcriptomic Data

For data analyses, the raw data files in Thermo.RAW format obtained in the profile mode were first centroided by conversion into .mzML format using ProteoWizard (Kessner et al., 2008) with peak picking filter applied. Features were detected and a pre-processed data table was created using the program, MZmine 2.53 (Pluskal et al., 2010), data were normalized using average squared intensities available within the MZmine workflow (Katajamaa and Orešiè, 2005) to enable comparison between different samples and the peak areas were exported to a single combined data table containing 2,235 metabolomic features. Global changes in metabolome were visualized using UMAP (McInnes et al., 2018). Raw data (.mzML format) are publicly available at National Metabolomics Data Repository [NMDR; Sud et al. (2016)]. A *t*-test was carried out to determine the significantly changed metabolites (*P* < 0.05) in pollen from the hydration or germination stage when compared with the mature pollen stage. The resultant subset of features that showed significant differences were annotated using “Functional Analysis” module of MetaboAnalyst v5.0 (Pang et al., 2021). Notably, for the untargeted metabolome analysis, as the annotated metabolites for the pathway enrichment analysis were predicted using computational algorithms (Li et al., 2013), the content of a specific metabolite needs to be manually verified if it is not provided in our already verified list. Pathway enrichment analysis was performed using the “Pathway Analysis” module of MetaboAnalyst v5.0. A differentially regulated gene list was directly retrieved from supplementary materials of published transcriptomic data by Wang et al. (2008). The differentially regulated genes and the consequential metabolites during the transition from mature to hydrated pollen, and from hydrated to germinated pollen, were integrated using the “Joint Pathway Analysis” module in MetaboAnalyst v5.0 with pathway database selection of “Metabolic pathways (integrated).” “Hypergeometric Test” was used for the enrichment analysis, “Degree Centrality” was selected for the topology analysis, and “Combine *p*-values (pathway-level)” was set as the integration method.

### Statistical Analysis

The Shapiro-Wilk test was used to test the normality of data. For the data that passed the normal distribution test, one-way ANOVA followed by multiple comparison tests (Fisher’s LSD method) was used for the difference comparisons among multiple subjects. For the data of metabolites (glucose, raffinose, stearic acid, oleic acid, IAA, and quercitin) non-normally distributed, the Kruskal-Wallis ANOVA followed by multiple comparison tests (Dunn’s test) was used. All statistical analysis was performed using OriginPro 2021b software (OriginLab Corporation, Northampton, MA, United States).

## Results

### Pollen Germination Stages and Statistical Analysis

**FIGURE 1:**
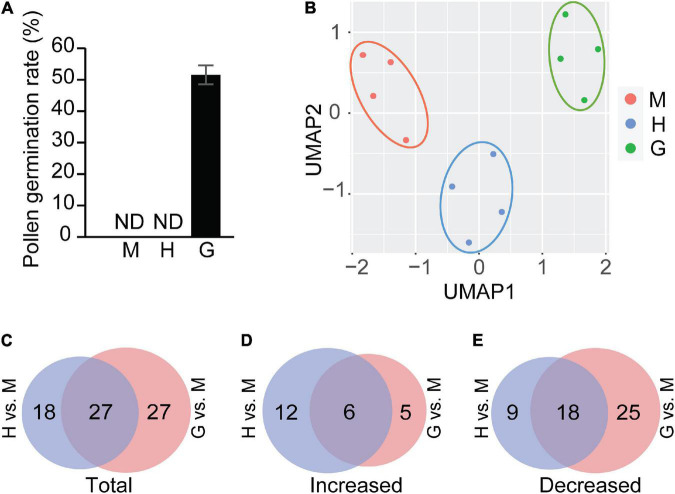
Pollen germination and statistical analysis of metabolites. **(A)** *In vitro* pollen germination assay for mature pollen (M), hydrated pollen (H), and germinated pollen (G). ND: not detected. The means were calculated from multiple repeats (±SE, *n* = 6), with over 420 pollen grains/tubes counted in total. **(B)** UMAP analysis of mature pollen(M), hydrated pollen (H), and germinated pollen (G). **(C)** Venn diagram showing total differential accumulated metabolites in hydrated pollen vs. in mature pollen (H vs. M) and those in hydrated pollen vs. in mature pollen (G vs. M). **(D)** Venn diagram showing increased metabolites in hydrated pollen vs. in mature pollen (H vs. M) and those in hydrated pollen vs. in mature pollen (G vs. M). **(E)** Venn diagram showing decreased metabolites in hydrated pollen vs. in mature pollen (H vs. M) and those in hydrated pollen vs. in mature pollen (G vs. M). Detailed compound information used in the Venn diagrams of panels **(C–E)** was shown in Supplementary Table 1.

Pollen germination rates were initially evaluated using a light microscope. Consistent with previous observations (Wang et al., 2008), no pollen from mature or hydrated stages was in a germinated form, while ∼52% of observed pollen germinated in the germinated pollen stage (Figure 1A). Three different chromatographic methods were used, including hydrophilic interaction chromatography (HILIC), reverse phase chromatography with a C18 column, and lipidomic profiling using a specific reverse phase C8 column. This ensured an extensive coverage of compounds and captured core central and specialized metabolites along with several lipid classes. To obtain a global overview of the metabolomic data, a UMAP (Uniform Manifold Approximation and Projection) analysis (McInnes et al., 2018), was performed to accommodate dimensionality reduction relative to PCA (Principal Component Analysis), and to visualize the data (Figure 1B). The four biological replicates for mature, hydrated, and germinated pollen were each clustered together and the clusters were easily distinguished along the UMAP1 dimension. The hydration stage was separated from the mature and germination stages as determined by the second UMAP dimension, suggesting many peak features were uniquely present and/or accumulated at distinct levels in this stage.

After determining the significant changes that occurred in metabolite levels (*P* < 0.05) between mature and hydrated pollen, and between mature and germinated pollen, we noted the significantly increased or decreased metabolites in each pair, with verified identities presented in Supplementary Table 1. Compared to mature pollen, a total of 45 and 54 metabolites were significantly altered in hydrated and germinated pollen, respectively. Of these, 27 of the same metabolites were altered in both hydrated and germinated pollen groups (Figure 1C). Where metabolite levels increased, the majority (18/23) of metabolites were found in hydrated pollen (Figure 1D), suggesting a potential role in metabolic activation before pollen tube emergence (Kim et al., 2019). Where metabolite levels decreased, 25 metabolites were found only in germinated pollen, while nine were found only in the hydrated pollen (Figure 1E).

### Differential Metabolic Pathway Enrichment Analysis

**FIGURE 2:**
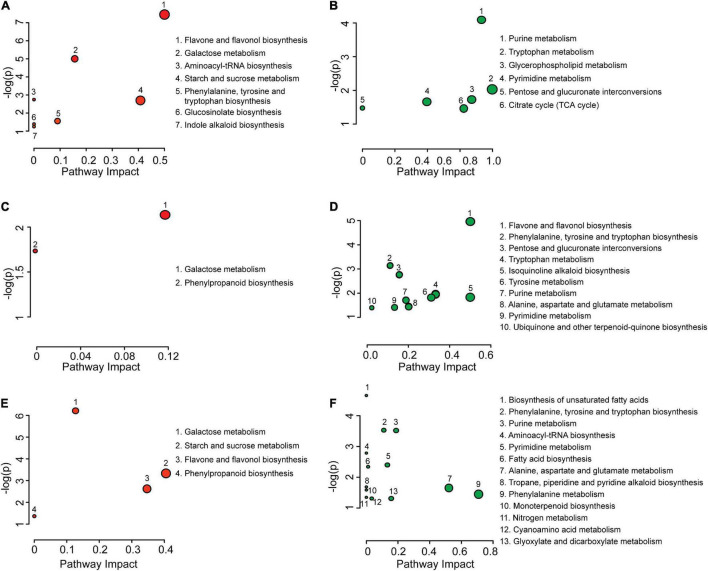
Pathway enrichment analysis of differential metabolites from mature pollen, hydrated pollen, and germinated pollen. Pathway enrichment analysis of increased **(A)** and decreased **(B)** metabolites between mature pollen and hydrated pollen. Pathway enrichment analysis of increased **(C)** and decreased **(D)** metabolites between hydrated pollen and germinated pollen. Pathway enrichment analysis of increased **(E)** and decreased **(F)** metabolites between mature pollen and germinated pollen. For all enrichment analysis *P* < 0.05, the circle size corresponds to the impact of the metabolic pathway, which evaluates the ratio of matched metabolites to overall metabolites in each pathway during the pathway topology analysis.

The metabolite differences were subjected to pathway enrichment analysis (Figure 2) where “enrichment” refers to an overrepresentation of the number of metabolites within a pathway that are coordinately elevated or reduced in level. During the transition from mature pollen to hydrated pollen, seven metabolic pathways were overrepresented in metabolites with elevated levels. Six metabolic pathways were overrepresented in metabolites with decreased levels (Figures 2A,B). During the transition from hydrated pollen to germinated pollen, two metabolic pathways were overrepresented in metabolites with elevated levels and ten pathways were overrepresented in metabolites with reduced levels (Figures 2C,D). When mature and germinated pollen metabolites were compared, four metabolic pathways were overrepresented in metabolites with elevated levels and thirteen metabolic pathways were overrepresented in metabolites with lowered levels (Figures 2E,F). Specifically, starch and sucrose metabolism, galactose metabolism, and flavone and flavonol biosynthetic pathways had disproportionate numbers of metabolites with increased levels in both hydrated and germinated pollen. By contrast, purine and pyrimidine metabolic pathways had more metabolites that were lowered in levels in both hydrated and germinated pollen. Interestingly, the quantity of metabolites from two overrepresented pathways increased during the transition from mature pollen to hydrated pollen, and then decreased during the transition from hydrated pollen to germinated pollen. One of the two pathways was the biosynthesis of flavone and flavonol, and the other was biosynthesis of phenylalanine, tyrosine and tryptophan. Both pathways had over-represented numbers of elevated metabolites in the hydrated pollen compared to the other two stages, suggesting their potential roles in metabolic activation for pollen tube emergence. Detailed information on pathway enrichment analysis can be found in Supplementary Table 2.

### Integrative Analysis of Transcriptomics and Metabolomics

To obtain a deeper understanding, we performed a multi-omics analysis that integrated the current metabolomic data with previously published expression levels from transcriptomics (Wang et al., 2008). As shown in Table 1, two pathways contained an overrepresented number of metabolites and genes that were either elevated or lowered in level in hydrated pollen compared to mature pollen. Three pathways contained an overrepresented number of genes and metabolites with either heightened or reduced levels in germinated pollen when compared to hydrated pollen. The list of compounds and genes from the joint analysis can be found in Supplementary Table 3. To investigate whether metabolic changes were associated with transcriptional changes, we performed a pathway enrichment analysis using the previously published transcriptome data. Unexpectedly, few of the identified pathways (e.g., linoleic acid metabolism, glycerophospholipid metabolism and purine metabolism) from our joint analysis data were significantly enriched in transcriptomics data (Supplementary Table 4). This is not surprising as disconcordance between protein and transcript levels is well-documented [Fernie and Stitt, 2012; summarized in Allen (2016)]; however, the results imply that integrative analysis can provide more insight than transcriptomics alone. Consistent with pathway enrichment analysis based only on metabolomics data, we found that starch and sucrose metabolism as well as galactose metabolism were over-represented in both hydrated and germinated pollen in the joint pathway analysis, suggesting that the compounds and enzymes related to soluble carbohydrate metabolism and cell-wall modifications were active during both pollen hydration and germination. In the published transcriptome data (Wang et al., 2008), *AtSUC3 (At2g02860)* and *AtSUC9 (At5g06170)*, which encode plasma-membrane localized sucrose transporters, had increased expression in germinated pollen. *AtSIP2 (At3g57520)*, which encodes a raffinose synthase, had elevated expression in germinated pollen. The gene expression levels were consistent with increased sucrose and raffinose that accumulated in germinated pollen (Supplementary Table 1). To investigate whether starch is altered during pollen germination, we performed starch staining using iodine solution on mature, hydrated, and germinated pollen. No clear differences were observed in the starch stains from the pollen in any of the three stages (Supplementary Figure 2), consistent with a previous report that no evidence of starch was detected in the mature *Arabidopsis* pollen grains after staining with iodine containing solution (Regan and Moffatt, 1990). As shown in the joint analysis (Supplementary Table 3), *AtUGE1 (At1g12780)*, which encodes a UDP-glucose epimerase, was expressed to a higher degree in germinated pollen, and *AtUGE3 (At1g63180)* was also elevated in both hydrated and germinated pollen. Plant UGEs are important to the regulation of cell wall carbohydrate biosynthesis (Rösti et al., 2007), which may contribute to the heightened metabolite levels in galactose metabolism from hydrated and germinated pollen.

**TABLE 1:** Joint pathway analysis of transcriptomics and metabolomics in hydrated pollen and germinated pollen.

	Enriched pathway	Total. compound	Hits. compound	Total. gene	Hits. gene	*P*	Impact
Up in hydrated pollen	Starch and sucrose metabolism	22	3	35	4	<0.001	0.55
	Galactose metabolism	27	5	24	1	<0.001	0.36
Down in hydrated pollen	Linoleic acid metabolism	63	7	78	1	0.042	0.26
	Pentose and glucuronate interconversions	16	2	14	1	0.032	0.21
Up in germinated pollen	Starch and sucrose metabolism	22	1	35	9	0.005	0.48
	Galactose metabolism	27	2	24	3	0.035	0.54
	Glutathione metabolism	26	1	24	8	0.003	0.49
Down in germinated pollen	Pentose and glucuronate interconversions	16	4	14	6	<0.001	0.79
	Glycerophospholipid metabolism	37	3	46	10	0.006	0.61
	Purine metabolism	63	6	78	10	0.03	0.66

*Total represents the total number of compounds/genes in each pathway; hits represents the number of compounds/genes that were significantly changed within each pathway.*

### Overview of All Differentially Accumulated Metabolites Among Mature, Hydrated, and Germinated Pollen

**FIGURE 3:**
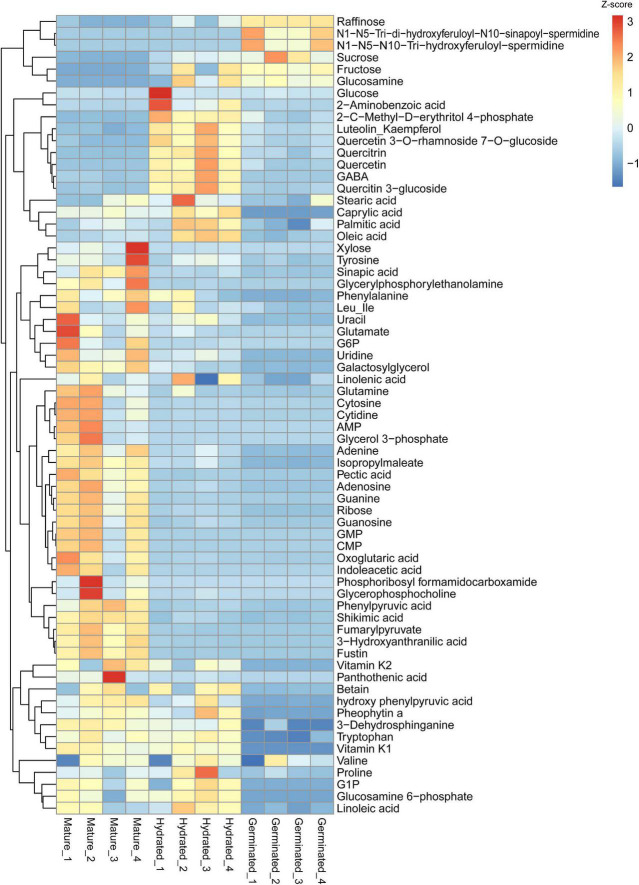
Heatmap showing the identified metabolite differences among mature pollen, hydrated and germinated pollen. Heatmap was created using raw data in intensity converted to z-scores for each metabolite using the “pheatmap” package (Kolde, 2019) in R. The scale bar represents the distance of the raw intensity away from the group mean in units of the standard deviation for a given metabolite. Z-score is negative when the raw intensity is below the mean, positive when above.

To give an overall comparison of metabolites among mature, hydrated, and germinated pollen, we summarized intensities in a heatmap of all manually verified metabolites (Supplementary Table 5) that passed the statistical threshold during metabolomics analysis (Figure 3). Four major clusters of metabolites were identified. The first cluster was enriched with metabolites accumulating in germinated pollen (e.g., raffinose, glucosamine). The second cluster contained metabolites enriched in hydrated pollen, including 2-aminobenzoic acid and oleic acid, and the third cluster represented metabolites elevated in mature pollen (e.g., xylose and fustin). The fourth cluster contained metabolites reduced in germinated pollen such as vitamin K2 and linoleic acid. All lipid species (including both neutral and polar lipids) were compared in a second heatmap (Supplementary Figure 1) and were mostly elevated in mature pollen but reduced in germinated pollen.

### Carbon-Related Metabolites Were Differentially Altered in Hydrated and Germinated Pollen

**FIGURE 4:**
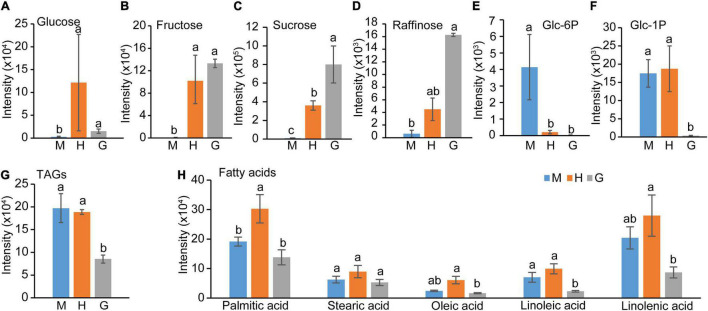
Central carbon intermediates changes among mature pollen (M), hydrated (H) and germinated (G) pollen. **(A)** Glucose, **(B)** Fructose, **(C)** Sucrose, **(D)** Raffinose, **(E)** Glucose-6-phosphate, **(F)** Glucose-1-phosphate, **(G)** total triacylglycerides, **(H)** Fatty acids content in mature, hydrated, and germinated pollen. The means (± SE, *n* = 4) were plotted. The statistically significant differences among mature, hydrated, and germinated pollen for a particular compound were represented by different letters (*P* < 0.05).

Sugar metabolism and fatty acid metabolism were among the enriched pathways during pollen hydration and germination. The normalized intensities of soluble sugars, fatty acids, lipids, and their adducts were retrieved from the metabolomic data (Supplementary Table 5). As shown in Figures 4A,B, glucose and fructose content were significantly elevated in hydrated pollen but unchanged in germinated pollen. By contrast, sucrose and raffinose content continued to increase during the transition from mature to hydrated to germinated pollen (Figures 4C,D). In contrast, glucose 6-phosphate, a precursor and product of sucrose metabolism, sharply decreased from mature to hydrated pollen (Figure 4E). Glucose 1-phosphate, which is also closely related to sucrose, dramatically decreased from hydrated to germinated pollen (Figure 4F). There was significantly reduced stored lipid in the form of triacylglycerol (TAG) in germinated pollen after hydration (Figure 4G). *AtDGAT (At2g19450)*, which encodes the key diacylglycerol acyltransferase for TAG biosynthesis (Routaboul et al., 1999), was reduced in expression during pollen germination (Supplementary Table 3). Other lipid species (e.g., diacylglycerol) were also reduced during pollen germination (Supplementary Figure 1). Non-esterified fatty acids including linoleic acid and linolenic acid, the two most abundant unsaturated fatty acids found in *Arabidopsis* flowers (Li-Beisson et al., 2009), decreased in germinated pollen after pollen hydration (Figure 4H). Furthermore, palmitic acid, oleic acid, and linolenic acid content peaked in hydrated pollen before decreasing during pollen germination (Figure 4H), suggesting they may be involved in metabolic activation during pollen hydration. The lack of the three major fatty acids (palmitic acid, oleic acid and linolenic acid) in the pollen coat is known to result in rapid dehydration of rice pollen grains (Xue et al., 2018). Taken together, the data suggests that lipids stored in mature pollen catabolized to support the energy-demanding pollen germination process. Saccharides, including sucrose and raffinose, accumulated as pollen germination progressed, likely due to the carbon supply from the external sucrose-rich medium provided during *in vitro* germination.

### Amino Acids, Hormone, and Flavonoids Changes in Hydrated and Germinated Pollen

**FIGURE 5:**
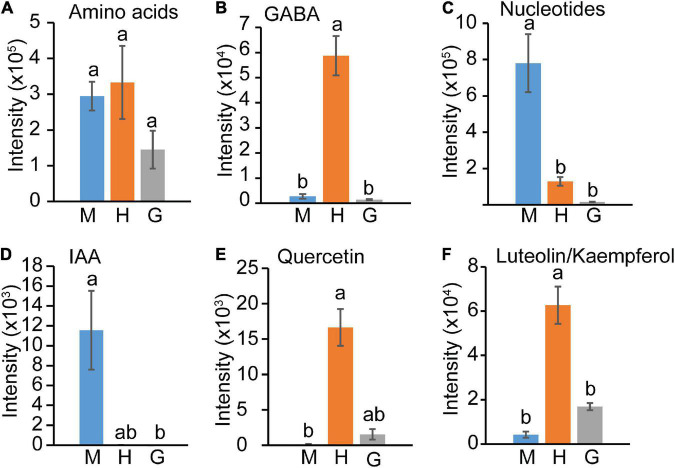
Amino acids, hormone, and flavonoids changes among mature pollen (M), hydrated (H) and germinated (G) pollen. **(A)** Amino acids, **(B)** GABA, **(C)** Nucleotides, **(D)** IAA, **(E)** Quercetin, **(F)** Luteolin/Kaempferol content in mature, hydrated, and germinated pollen. The means (±SE, *n* = 4) were plotted. The statistically significant differences among mature, hydrated, and germinated pollen for a particular compound were represented by different letters (*P* < 0.05).

In contrast to central carbon intermediates, the levels of detected amino acids (Val, Tyr, Trp, Pro, Phe, Leu/Ile, Gln, Glu; Supplementary Table 5) did not change significantly within the three pollen stages (Figure 5A). γ-aminobutyric acid (GABA) showed a special pattern (Figure 5B), increasing in hydrated pollen followed by a sharp decrease to base level in germinated pollen. The levels of all identified compounds involved in nucleotide metabolism, including adenosine monophosphate (AMP), guanosine monophosphate (GMP), cytidine monophosphate (CMP), guanosine, adenosine, uridine, cytosine, cytidine, guanine, adenine, uracil (Figure 3), significantly decreased from mature pollen to hydrated pollen and remained low in germinated pollen (Figure 5C). Decreases in uracil and uridine in transition from mature pollen to germinated pollen suggest that the mature pollen grain is primed for rapid translation and protein synthesis upon germination. Indoleacetic acid (IAA) content also declined significantly from mature to hydrated pollen and remained at a low level in germinated pollen (Figure 5D). GABA, quercetin and the flavonoids luteolin/kaempferol sharply increased in hydrated pollen before returning to a low level in germinated pollen (Figures 5E,F) and may be related to metabolic activation for pollen tube emergence.

## Discussion

Before anthesis, pollen undergoes a maturation process which includes dehydration and accumulation of storage reserves. The degree of dehydration is highly correlated with carbohydrates and lipid levels in mature pollen (Shi and Yang, 2010). However, the form of carbohydrates and dehydration status in mature pollen grains varies among plant species (Pacini, 1996; Pacini et al., 2006). Grass pollen grains generally contain a low level of water, a high level of starch, and are short-lived (Shi and Yang, 2010; Kim et al., 2019) compared to dicot pollen grains (*Arabidopsis*), which accumulate a high level of lipids instead of starch (Kuang and Musgrave, 1996; Ischebeck, 2016). It has been well-documented that *Arabidopsis* mature pollen is virtually starch-free (Regan and Moffatt, 1990; Kuang and Musgrave, 1996; Tang et al., 2009; Streb and Zeeman, 2012), which is consistent with the lack of clear starch stains in mature, hydrated, and germinated pollen (Supplementary Figure 2). In addition to changes in composition, pollen metabolism responds rapidly upon interacting with stigma cells including translation of mRNA and activation of stored enzymes to engage pollen hydration (Shi and Yang, 2010). Compared to those in mature pollen grain, metabolites from a number of central carbon biosynthetic pathways, e.g., purine and pyrimidine metabolism and fatty acids biosynthesis, were significantly reduced in hydrated pollen or germinated pollen, possibly due to increased turnover (Figures 2B,D,F), and consistent with the use of fatty acids (Figure 4H) and nucleotides (Figure 5C)/purines (Figure 3) that are building blocks for growth and were detected at higher levels in mature pollen relative to hydrated or germinated pollen.

Although *Arabidopsis* mature pollen is stored with lipid and carbohydrate reserves, the amount is insufficient to sustain rapid pollen tube growth given its small pollen grain size/volume (De Storme et al., 2013) and additional supplies of sugars are needed during pollen tube growth (Reinders, 2016). This concept was supported with our observations from metabolomic data and the integration of metabolome and transcriptome. We found elevated levels of starch and sucrose metabolism during the transition from mature to hydrated pollen (Figure 2A and Table 1) and from hydrated to germinated pollen (Figure 2C and Table 1), suggesting soluble carbohydrate metabolism is most active in germinated followed by hydrated and mature pollen, respectively. The elevated soluble carbohydrate metabolism can contribute to elevated sucrose and raffinose in germinated pollen (Figures 4C,D). The elevated demand for sucrose during pollen germination can be accommodated by expression of sucrose transporters like *AtSUC1* (*At1g71880*), which when absent result in a compromised pollen germination phenotype in mutants without affecting fatty acid content (Sivitz et al., 2008). By contrast, both monosaccharides (glucose and fructose; Figures 4A,B) and di- and trisaccharides (sucrose and raffinose; Figures 4C,D) contributed to elevated carbohydrate levels in hydrated pollen. Interestingly, there were significantly higher levels of glucose and fructose in hydrated pollen and maintained at a similar level in germinated pollen after hydration. Based on targeted metabolite quantifications, elevated glucose and fructose were also found in hydrated *Arabidopsis* pollen compared to mature pollen (Wang et al., 2022).

Monosaccharides, including glucose and fructose, do not support *in vitro* pollen germination of *Arabidopsis*, while di- and trisaccharides, including sucrose and raffinose, can support *in vitro* pollen germination of *Arabidopsis* (Hirsche et al., 2017). Because only sucrose was supplied in the *in vitro* germination medium, the elevated glucose and fructose level in hydrated pollen were likely due to sucrose hydrolysis. Plasma-membrane localized sucrose transporters, such as AtSUC1 (Stadler et al., 1999; Sivitz et al., 2008), and alkaline/neutral invertases, such as A/N-InvH (*At3g05820*) that hydrolyze sucrose into fructose and glucose, are highly expressed in pollen (Wang et al., 2008) and *atsuc1* and *invh* mutants exhibit reduced pollen germination and fewer seeds per silique, respectively (Sivitz et al., 2008; Battaglia et al., 2017). When cell-wall invertase activity has been reduced via RNA-interference or an invertase inhibitor, pollen germination rates and seed sets are reduced in both *A. thaliana* and *N. tabacum* (Hirsche et al., 2009). Soluble carbohydrates, especially glucose, are potent signaling molecules involved in many aspects of plant growth (Rolland et al., 2006); thus, the accumulated glucose and fructose in hydrated pollen may initiate signaling events that result in metabolic activation before pollen tube emergence. Increased glucose levels were not observed in germinated pollen, suggesting pollen metabolism limits glucose levels to avoid pollen tube growth inhibition known to occur at high glucose concentrations (Rottmann et al., 2018).

In our results, almost all lipid species accumulated to high levels in mature pollen and were subsequently reduced in germinated pollen (Supplementary Figure 1), consistent with the lipid accumulation pattern that was observed in tobacco pollen (Dorne et al., 1988). Neutral lipids including TAG in tobacco pollen were reduced during 6 h of pollen germination, similar to lipid bodies in olive pollen after 7 h of pollen germination (Rodriguez-Garcia et al., 2003). Another study detailed changes in neutral and polar lipid fractions over olive pollen germination (Hernández et al., 2020). Interestingly, they found the fatty acid composition from total lipids was significantly altered, although the TAG content remained unchanged during the first 6 h of germination (Hernández et al., 2020). These results indicate lipid dynamic changes are stage- and species-dependent. As widely reported, an external supply of sucrose is required for *in vitro Arabidopsis* pollen germination, though the presence or absence of sugars had little impact on *in vitro* olive pollen germination or pollen tube growth rates (Zienkiewicz et al., 2013), possibly suggesting lipid reserves are the primary carbon source for olive pollen germination without significant requirement for sugars. On the other hand, a different pattern in sugar accumulation (relative to our study) was reported during tobacco pollen germination (Rotsch et al., 2017). To be specific, sucrose decreased sharply after pollen rehydration although remaining low during pollen germination, while fructose progressively increased as pollen germination proceeded. Tobacco pollen can also germinate without any sugars (Rotsch et al., 2017), suggesting that lipid and sucrose reserves in tobacco pollen are sufficient for subsequent germination. Thus, changes in metabolite levels, such as sugars and lipids, over the pollen germination stages are highly species-dependent and will require further investigations to understand the differences in mechanisms underlying *in vitro* pollen germination.

The GABA content peaked in hydrated pollen (Figure 5B). In plants, GABA levels are regulated by stress, signaling, energy production and play a major role in balancing carbon/nitrogen metabolism by linking amino acid metabolism and the TCA cycle through the GABA shunt (Fait et al., 2008). Exogenous GABA stimulates *Arabidopsis* pollen tube growth at low concentrations but inhibits pollen tube growth at high concentrations during *in vitro* pollen germination (Palanivelu et al., 2003), likely through GABA-gated aluminum-activated malate transporter (ALMT) (Ramesh et al., 2015). The mutant of the POP2 gene, which encodes a transaminase that degrades GABA, accumulates a high level of GABA and the pollen tube growth of *pop2* is arrested (Palanivelu et al., 2003), suggesting the GABA level plays a critical role in pollen tube growth/signaling and similar GABA patterns in lily and tobacco pollen germination (Obermeyer et al., 2013; Rotsch et al., 2017) may suggest conserved roles in development across some species.

Auxin is one of the most important hormones to promote cell division and elongation in plants (Zhao, 2010). Auxin plays a critical role in *Arabidopsis* pollen maturation (Cecchetti et al., 2008; Salinas-Grenet et al., 2018), but external auxin treatment reduced *in vitro* pollen germination rate of *Arabidopsis* (Ding et al., 2012). Our observations that IAA is accumulated at high levels in mature pollen, while barely detectable in hydrated pollen and germinated pollen (Figure 5D) is consistent with the prior descriptions. However, external IAA can stimulate *in vitro* pollen tube growth of *Nicotiana tabacum* (Chen and Zhao, 2008) and *Torenia fournieri* (Wu et al., 2008), suggesting the role of auxin during pollen tube growth is species-dependent.

The role of flavonoids during pollen germination also varies among different plant species. A male sterile phenotype of flavonoid-deficient mutant was observed in maize (Coe et al., 1981) and petunia (Ylstra et al., 1994), but *Arabidopsis* plants that are deficient in flavonoid biosynthesis appear to be fully fertile (Burbulis et al., 1996). As common flavonoids in plants, quercetin and luteolin/kaempferol accumulate to high levels in hydrated pollen (Figures 5E,F), flavone and flavonol biosynthesis was engaged and resulted in elevated metabolite levels during pollen hydration (Figure 2A). Our results support flavonoid involvement in the onset of pollen germination, but its role on pollen fertility may be limited in *Arabidopsis.*

As already mentioned, the germinated pollen samples object of this study consisted of both non-germinated and germinated pollen, with an overall germination rate of ∼52%, comparable to previously published rates for *Arabidopsis* [39% in Sivitz et al. (2008); 58% in Qin et al. (2009); 48.9% in Hirsche et al. (2017)]. Pollen germination is a consequence of both the rate of hydration and the speed at which the pollen tube tip is able to accomplish tube emergence (Firon et al., 2012). The fact that a substantial number of pollen grains failed to germinate *in vitro* indicates issues connected to the hydration process in non-germinable pollen grains. Although it would be possible to largely separate pollen tubes from non-germinated pollen grains using a 50-μm nylon mesh (Wang et al., 2008), we faced the impossibility of sufficiently remove these non-germinable pollen grains from the hydrated pollen samples on large scale. Thus, what we called germinated pollen samples were mixed samples (germinated and non-germinated) in order to compare them to the mixed samples of hydrated pollen (germinable and non-germinable) and to already published results with similar germination rates. Previously reported metabolomic (Obermeyer et al., 2013; Fragallah et al., 2018) and transcriptomic (Qin et al., 2009) studies on germinated pollen followed a really similar approach, thus, increasing our confidence on the results obtained.

To conclude, a comprehensive metabolome analysis during *Arabidopsis* pollen germination in combination with published transcriptome revealed a complicated metabolic pathway network in support of pollen hydration and germination. The detailed analyses of carbohydrates and fatty acids indicated their roles in carbon metabolism that varied in mature, hydrated, and germinated pollen stages and a unique set of metabolites were identified to accumulate in the hydrated pollen stage, but were barely accumulated in other two stages.

## Data Availability Statement

Raw and pre-processed metabolomic data is available at the NIH Common Fund’s National Metabolomics Data Repository (NMDR) website, the Metabolomics Workbench, https://www.metabolomicsworkbench.org where it has been assigned Study ID ST002060. The data can be accessed directly via its Project doi: 10.21228/M8570V.

## Author Contributions

JW and L-QC conceived and designed the experiments. JW and SK conducted the experiments and performed the metabolomics analysis. DA supervised the metabolomics analysis and edited the manuscript. JW, SK, and L-QC wrote the manuscript. All authors contributed to the article and approved the submitted version.

## Conflict of Interest

The authors declare that the research was conducted in the absence of any commercial or financial relationships that could be construed as a potential conflict of interest.

## Publisher’s Note

All claims expressed in this article are solely those of the authors and do not necessarily represent those of their affiliated organizations, or those of the publisher, the editors and the reviewers. Any product that may be evaluated in this article, or claim that may be made by its manufacturer, is not guaranteed or endorsed by the publisher.

## Supplementary Material

Supplementary Figure 1 Heatmap showing the identified lipid species differences among mature pollen, hydrated and germinated pollen. Heatmap was carried out on raw intensity data converted to z-scores for each metabolite using the “pheatmap” package in R. The scale bar represents the distance between raw intensity and the population mean in units of the standard deviation for a given metabolite. Z-score is negative when the raw intensity is below the mean, positive when above. DG, diacylglycerol; TG, triacylglycerol; PA, phosphatidic acid; PC, phosphatidylcholine; PI, phosphatidylinositol; PG, phosphatidylglycerol; PE, phosphatidylethanolamine; LPC, lysophosphatidylcholine; LPE, lysophosphatidylethanolamine.

Supplementary Figure 2 Starch staining of mature, hydrated, and germinated pollen. Mature, hydrated, and germinated pollen collected from 50 flowers were stained using iodine solution. No clear starch stains (dark purple) were observed from mature, hydrated, and germinated pollen.

## References

[B1] Allen D. K. (2016). Quantifying plant phenotypes with isotopic labeling & metabolic flux analysis. *Curr. Opin. Biotechnol.* 37 45–52. 10.1016/j.copbio.2015.10.002 26613198

[B2] Battaglia M. E. Martin M. V. Lechner L. Martínez-Noël G. M. A. Salerno G. L. (2017). The riddle of mitochondrial alkaline/neutral invertases: a novel *Arabidopsis* isoform mainly present in reproductive tissues and involved in root ROS production. *PLoS One* 12:e0185286. 10.1371/journal.pone.0185286 28945799 PMC5612693

[B3] Burbulis I. E. Iacobucci M. Shirley B. W. (1996). A null mutation in the first enzyme of flavonoid biosynthesis does not affect male fertility in *Arabidopsis*. *Plant Cell* 8 1013–1025. 10.1105/tpc.8.6.1013 8672888 PMC161155

[B4] Castro A. J. Clément C. (2007). Sucrose and starch catabolism in the anther of *Lilium* during its development: a comparative study among the anther wall, locular fluid and microspore/pollen fractions. *Planta* 225 1573–1582. 10.1007/s00425-006-0443-5 17123100

[B5] Cecchetti V. Altamura M. M. Falasca G. Costantino P. Cardarelli M. (2008). Auxin regulates *Arabidopsis* anther dehiscence, pollen maturation, and filament elongation. *Plant Cell* 20 1760–1774. 10.1105/tpc.107.057570 18628351 PMC2518247

[B6] Chen D. Zhao J. (2008). Free IAA in stigmas and styles during pollen germination and pollen tube growth of *Nicotiana tabacum*. *Physiol. Plant.* 134 202–215. 10.1111/j.1399-3054.2008.01125.x 18485059

[B7] Chen S. Zhong W. Liu M. Xie Z. Wang H. (2008). Pollen grain germination and pollen tube growth in pistil of rice. *Rice Sci.* 15 125–130. 10.1016/S1672-6308(08)60030-X

[B8] Coe E. H. Mccormick S. M. Modena S. A. (1981). White pollen in maize. *J. Hered.* 72 318–320. 10.1093/oxfordjournals.jhered.a109514

[B9] De Storme N. Zamariola L. Mau M. Sharbel T. F. Geelen D. (2013). Volume-based pollen size analysis: an advanced method to assess somatic and gametophytic ploidy in flowering plants. *Plant Reprod.* 26 65–81. 10.1007/s00497-012-0209-0 23686220

[B10] Ding Z. J. Wang B. J. Moreno I. Duplakova N. Simon S. Carraro N. (2012). ER-localized auxin transporter PIN8 regulates auxin homeostasis and male gametophyte development in *Arabidopsis*. *Nat. Commun.* 3:941. 10.1038/ncomms1941 22760640

[B11] Dorne A.-J. Kappler R. Kristen U. Heinz E. (1988). Lipid metabolism during germination of tobacco pollen. *Phytochemistry* 27 2027–2031. 10.1016/0031-9422(88)80090-6

[B12] Fait A. Fromm H. Walter D. Galili G. Fernie A. R. (2008). Highway or byway: the metabolic role of the GABA shunt in plants. *Trends Plant Sci.* 13 14–19. 10.1016/j.tplants.2007.10.005 18155636

[B13] Fernie A. R. Stitt M. (2012). On the discordance of metabolomics with proteomics and transcriptomics: coping with increasing complexity in logic, chemistry, and network interactions scientific correspondence. *Plant Physiol.* 158 1139–1145. 10.1104/pp.112.193235 22253257 PMC3291261

[B14] Fiebig A. Mayfield J. A. Miley N. Chau S. Fischer R. Preuss D. (2000). Alterations in CER6, a gene identical to CUT1, differentially affect long-chain lipid content on the surface of pollen and stems. *Plant Cell* 12 2001–2008. 10.1105/tpc.12.10.2001 11041893 PMC149136

[B15] Firon N. Nepi M. Pacini E. (2012). Water status and associated processes mark critical stages in pollen development and functioning. *Ann. Bot.* 109 1201–1214. 10.1093/aob/mcs070 22523424 PMC3359924

[B16] Fragallah S. Wang P. Li N. Chen Y. Lin S. Z. (2018). Metabolomic analysis of pollen grains with different germination abilities from two clones of Chinese fir (*Cunninghamia lanceolata* (lamb) hook). *Molecules* 23:3162. 10.3390/molecules23123162 30513683 PMC6321011

[B17] Goetz M. Guivarch A. Hirsche J. Bauerfeind M. A. Gonzalez M. C. Hyun T. (2017). Metabolic control of tobacco pollination by sugars and invertases. *Plant Physiol.* 173 984–997. 10.1104/pp.16.01601 27923989 PMC5291038

[B18] Goldman M. H. S. Goldberg R. B. Mariani C. (1994). Female sterile tobacco plants are produced by stigma-specific cell ablation. *EMBO J.* 13 2976–2984. 10.1002/j.1460-2075.1994.tb06596.x 8039494 PMC395185

[B19] Hafidh S. Honys D. (2021). Reproduction multitasking: the male gametophyte. *Annu. Rev. Plant Biol.* 72 581–614. 10.1146/annurev-arplant-080620-021907 33900787

[B20] Hernández M. L. Lima-Cabello E. Alché J. de D. Martínez-Rivas J. M. Castro A. J. (2020). Lipid composition and associated gene expression patterns during pollen germination and pollen tube growth in olive (*Olea europaea L.*). *Plant Cell Physiol.* 61 1348–1364. 10.1093/pcp/pcaa063 32384163 PMC7377348

[B21] Heslop-Harrison J. (1979). Aspects of the structure, cytochemistry and germination of the pollen of rye (*Secale cereale L.*). *Ann. Bot.* 44 1–47.

[B22] Hirsche J. Engelke T. Voller D. Gotz M. Roitsch T. (2009). Interspecies compatibility of the anther specific cell wall invertase promoters from *Arabidopsis* and tobacco for generating male sterile plants. *Theor. Appl. Genet.* 118 235–245. 10.1007/s00122-008-0892-2 18825361

[B23] Hirsche J. Fernandez J. M. G. Stabentheiner E. Grosskinsky D. K. Roitsch T. (2017). Differential effects of carbohydrates on *Arabidopsis* pollen germination. *Plant Cell Physiol.* 58 691–701. 10.1093/pcp/pcx020 28339807

[B24] Hummel J. Segu S. Li Y. Irgang S. Jueppner J. Giavalisco P. (2011). Ultra performance liquid chromatography and high resolution mass spectrometry for the analysis of plant lipids. *Front. Plant Sci.* 2:54. 10.3389/fpls.2011.00054 22629264 PMC3355513

[B25] Ischebeck T. (2016). Lipids in pollen – they are different. *Biochim. Biophys. Acta Mol. Cell Biol. Lipids* 1861 1315–1328. 10.1016/j.bbalip.2016.03.023 27033152

[B26] Johnson M. A. Harper J. F. Palanivelu R. Merchant S. S. (2019). A fruitful journey: pollen tube navigation from germination to fertilization. *Annu. Rev. Plant Biol.* 70 809–837. 10.1146/annurev-arplant-050718-100133 30822112

[B27] Johnson S. A. McCormick S. (2001). Pollen germinates precociously in the anthers of raring-to-go, an *Arabidopsis* gametophytic mutant. *Plant Physiol.* 126 685–695. 10.1104/pp.126.2.685 11402197 PMC111159

[B28] Johnson-Brousseau S. A. McCormick S. (2004). A compendium of methods useful for characterizing *Arabidopsis* pollen mutants and gametophytically- expressed genes. *Plant J.* 39 761–775. 10.1111/j.1365-313X.2004.02147.x 15315637

[B29] Kambhampati S. Aznar-Moreno J. A. Bailey S. R. Arp J. J. Chu K. L. Bilyeu K. D. (2021). Temporal changes in metabolism late in seed development affect biomass composition. *Plant Physiol.* 186 874–890. 10.1093/plphys/kiab116 33693938 PMC8195533

[B30] Katajamaa M. Orešič M. (2005). Processing methods for differential analysis of LC/MS profile data. *BMC Bioinformatics* 6:179. 10.1186/1471-2105-6-179 16026613 PMC1187873

[B31] Kessner D. Chambers M. Burke R. Agusand D. Mallick P. (2008). ProteoWizard: open source software for rapid proteomics tools development. *Bioinformatics* 24 2534–2536. 10.1093/bioinformatics/btn323 18606607 PMC2732273

[B32] Kim Y. J. Zhang D. B. Jung K. H. (2019). Molecular basis of pollen germination in cereals. *Trends Plant Sci.* 24 1126–1136. 10.1016/j.tplants.2019.08.005 31610991

[B33] Kolde R. (2019). *pheatmap: Pretty Heatmaps Version 1.0.12.* Available online at: https://rdrr.io/cran/pheatmap/ (accessed January 17, 2022).

[B34] Kuang A. Musgrave M. E. (1996). Dynamics of vegetative cytoplasm during generative cell formation and pollen maturation in *Arabidopsis thaliana*. *Protoplasma* 194 81–90. 10.1007/BF01273170 11540605

[B35] Li S. Z. Park Y. Duraisingham S. Strobel F. H. Khan N. Soltow Q. A. (2013). Predicting network activity from high throughput metabolomics. *PLoS Comput. Biol.* 9:e1003123. 10.1371/journal.pcbi.1003123 23861661 PMC3701697

[B36] Li-Beisson Y. Pollard M. Sauveplane V. Pinot F. Ohlrogge J. Beisson F. (2009). Nanoridges that characterize the surface morphology of flowers require the synthesis of cutin polyester. *Proc. Natl. Acad. Sci. U.S.A.* 106 22008–22013. 10.1073/pnas.0909090106 19959665 PMC2788479

[B37] McInnes L. Healy J. Melville J. (2018). UMAP: uniform manifold approximation and projection for dimension reduction. *arXiv* [preprint]. arXiv:1802.03426, 10.1093/bib/bbab008 33529337

[B38] Moon S. Jung K. H. (2020). First steps in the successful fertilization of rice and *Arabidopsis*: pollen longevity, adhesion and hydration. *Plants Basel* 9:956. 10.3390/plants9080956 32751098 PMC7465243

[B39] Obermeyer G. Fragner L. Lang V. Weckwerth W. (2013). Dynamic adaption of metabolic pathways during germination and growth of lily pollen tubes after inhibition of the electron transport chain. *Plant Physiol.* 162 1822–1833. 10.1104/pp.113.219857 23660836 PMC3729764

[B40] Pacini E. (1996). Types and meaning of pollen carbohydrate reserves. *Sex. Plant Reprod.* 9 362–366. 10.1007/BF02441957

[B41] Pacini E. Guarnieri M. Nepi M. (2006). Pollen carbohydrates and water content during development, presentation, and dispersal: a short review. *Protoplasma* 228 73–77. 10.1007/s00709-006-0169-z 16937057

[B42] Palanivelu R. Brass L. Edlund A. F. Preuss D. (2003). Pollen tube growth and guidance is regulated by POP2, an *Arabidopsis* gene that controls GABA levels. *Cell* 114 47–59. 10.1016/S0092-8674(03)00479-3 12859897

[B43] Pang Z. Q. Chong J. Zhou G. Y. Morais D. A. D. Chang L. Barrette M. (2021). MetaboAnalyst 5.0: narrowing the gap between raw spectra and functional insights. *Nucleic Acids Res.* 49 W388–W396. 10.1093/nar/gkab382 34019663 PMC8265181

[B44] Paupiere M. J. Muller F. Li H. J. Rieu I. Tikunov Y. M. Visser R. G. F. (2017). Untargeted metabolomic analysis of tomato pollen development and heat stress response. *Plant Reprod.* 30 81–94. 10.1007/s00497-017-0301-6 28508929 PMC5486769

[B45] Pluskal T. Castillo S. Villar-Briones A. Oresic M. (2010). MZmine 2: modular framework for processing, visualizing, and analyzing mass spectrometry-based molecular profile data. *BMC Bioinformatics* 11:395. 10.1186/1471-2105-11-395 20650010 PMC2918584

[B46] Qin Y. Leydon A. R. Manziello A. Pandey R. Mount D. Denic S. (2009). Penetration of the stigma and style elicits a novel transcriptome in pollen tubes, pointing to genes critical for growth in a pistil. *PLoS Genet.* 5:e1000621. 10.1371/journal.pgen.1000621 19714218 PMC2726614

[B47] Ramesh S. A. Tyerman S. D. Xu B. Bose J. Kaur S. Conn V. (2015). GABA signalling modulates plant growth by directly regulating the activity of plant-specific anion transporters. *Nat. Commun.* 6:7879. 10.1038/ncomms8879 26219411 PMC4532832

[B48] Regan S. M. Moffatt B. A. (1990). Cytochemical analysis of pollen development in wild-type *Arabidopsis* and a male-sterile mutant. *Plant Cell* 2 877–889. 10.1105/tpc.2.9.877 12354970 PMC159938

[B49] Reinders A. (2016). Fuel for the road – sugar transport and pollen tube growth. *J. Exp. Bot.* 67 2121–2123. 10.1093/jxb/erw113 27022182 PMC4809301

[B50] Rodriguez-Garcia M. I. M’rani-Alaoui M. Fernandez M. C. (2003). Behavior of storage lipids during development and germination of olive (*Olea europaea L.*) pollen. *Protoplasma* 221 237–244. 10.1007/s00709-002-0076-x 12802631

[B51] Rolland F. Baena-Gonzalez E. Sheen J. (2006). Sugar sensing and signaling in plants: conserved and novel mechanisms. *Annu. Rev. Plant Biol.* 57 675–709. 10.1146/annurev.arplant.57.032905.105441 16669778

[B52] Rösti J. Barton C. J. Albrecht S. Dupree P. Pauly M. Findlay K. (2007). UDP-Glucose 4-Epimerase isoforms UGE2 and UGE4 cooperate in providing UDP-Galactose for cell wall biosynthesis and growth of *Arabidopsis thaliana*. *Plant Cell* 19 1565–1579. 10.1105/tpc.106.049619 17496119 PMC1913733

[B53] Rotsch A. H. Kopka J. Feussner I. Ischebeck T. (2017). Central metabolite and sterol profiling divides tobacco male gametophyte development and pollen tube growth into eight metabolic phases. *Plant J.* 92 129–146. 10.1111/tpj.13633 28685881

[B54] Rottmann T. Fritz C. Sauer N. Stadler R. (2018). Glucose uptake via STP transporters inhibits in vitro pollen tube growth in a HEXOKINASE1-dependent manner in *Arabidopsis thaliana*. *Plant Cell* 30 2057–2081. 10.1105/tpc.18.00356 30120167 PMC6181011

[B55] Routaboul J. M. Benning C. Bechtold N. Caboche M. Lepiniec L. (1999). The TAG1 locus of *Arabidopsis* encodes for a diacylglycerol acyltransferase. *Plant Physiol. Biochem.* 37 831–840. 10.1016/S0981-9428(99)00115-1 10580283

[B56] Salinas-Grenet H. Herrera-Vasquez A. Parra S. Cortez A. Gutierrez L. Pollmann S. (2018). Modulation of auxin levels in pollen grains affects stamen development and anther dehiscence in *Arabidopsis*. *Int. J. Mol. Sci.* 19:2480. 10.3390/ijms19092480 30131475 PMC6164920

[B57] Shi D. Q. Yang W. C. (2010). “Pollen germination and tube growth,” in *Plant Developmental Biology-Biotechnological Perspectives*, eds Pua E. Davey M. (Berlin: Springer), 245–282.

[B58] Sivitz A. B. Reinders A. Ward J. M. (2008). *Arabidopsis* sucrose transporter AtSUC1 is important for pollen germination and sucrose-induced anthocyanin accumulation. *Plant Physiol.* 147 92–100. 10.1104/pp.108.118992 18359840 PMC2330317

[B59] Stadler R. Truernit E. Gahrtz M. Sauer N. (1999). The AtSUC1 sucrose carrier may represent the osmotic driving force for anther dehiscence and pollen tube growth in *Arabidopsis*. *Plant J.* 19 269–278. 10.1046/j.1365-313X.1999.00527.x 10476074

[B60] Streb S. Zeeman S. C. (2012). Starch metabolism in *Arabidopsis*. *Arabidopsis Book* 10:e0160. 10.1199/tab.0160 23393426 PMC3527087

[B61] Sud M. Fahy E. Cotter D. Azam K. Vadivelu I. Burant C. (2016). Metabolomics Workbench: an international repository for metabolomics data and metadata, metabolite standards, protocols, tutorials and training, and analysis tools. *Nucleic Acids Res.* 44 D463–D470. 10.1093/nar/gkv1042 26467476 PMC4702780

[B62] Tang L. Y. Nagata N. Matsushima R. Chen Y. Yoshioka Y. Sakamoto W. (2009). Visualization of plastids in pollen grains: involvement of FtsZ1?in pollen plastid division. *Plant Cell Physiol.* 50 904–908. 10.1093/pcp/pcp042 19282372

[B63] Wang J. Yu Y.-C. Li Y. Chen L.-Q. (2022). Hexose transporter SWEET5 confers galactose sensitivity to *Arabidopsis* pollen germination via a galactokinase. *Plant Physiol.*. 10.1093/plphys/kiac068 35188197 PMC9070816

[B64] Wang Y. Zhang W. Z. Song L. F. Zou J. J. Su Z. Wu W. H. (2008). Transcriptome analyses show changes in gene expression to accompany pollen germination and tube growth in *Arabidopsis*. *Plant Physiol.* 148 1201–1211. 10.1104/pp.108.126375 18775970 PMC2577266

[B65] Wolters-Arts M. Lush W. M. Mariani C. (1998). Lipids are required for directional pollen-tube growth. *Nature* 392 818–821. 10.1038/33929 9572141

[B66] Wu J. Z. Lin Y. Zhang X. L. Pang D. W. Zhao J. (2008). IAA stimulates pollen tube growth and mediates the modification of its wall composition and structure in *Torenia fournieri*. *J. Exp. Bot.* 59 2529–2543. 10.1093/jxb/ern119 18544613 PMC2423660

[B67] Xue Z. Y. Xu X. N. Zhou Y. Wang X. N. Zhang Y. C. Liu D. (2018). Deficiency of a triterpene pathway results in humidity-sensitive genic male sterility in rice. *Nat. Commun.* 9:604. 10.1038/s41467-018-03048-8 29426861 PMC5807508

[B68] Ylstra B. Busscher J. Franken J. Hollman P. C. H. Mol J. N. M. Vantunen A. J. (1994). Flavonols and fertilization in *petunia hybrida*: localization and mode of action during pollen tube growth. *Plant J.* 6 201–212. 10.1046/j.1365-313X.1994.6020201.x

[B69] Zhao Y. D. (2010). Auxin biosynthesis and its role in plant development. *Annu. Rev. Plant Biol.* 61 49–64. 10.1146/annurev-arplant-042809-112308 20192736 PMC3070418

[B70] Zienkiewicz A. Zienkiewicz K. Rejón J. D. Rodríguez-García M. I. Castro A. J. (2013). New insights into the early steps of oil body mobilization during pollen germination. *J. Exp. Bot.* 64 293–302. 10.1093/jxb/ers332 23132905 PMC3528035

