# Supplementary figures and images for: Comparative Metabolic Analysis Reveals a Metabolic Switch in Mature, Hydrated, and Germinated Pollen in Arabidopsis thaliana
Source: Front Plant Sci. 2022 May 18;13:836665. doi: 10.3389/fpls.2022.836665 (PMC9158543; doi:10.3389/fpls.2022.836665)

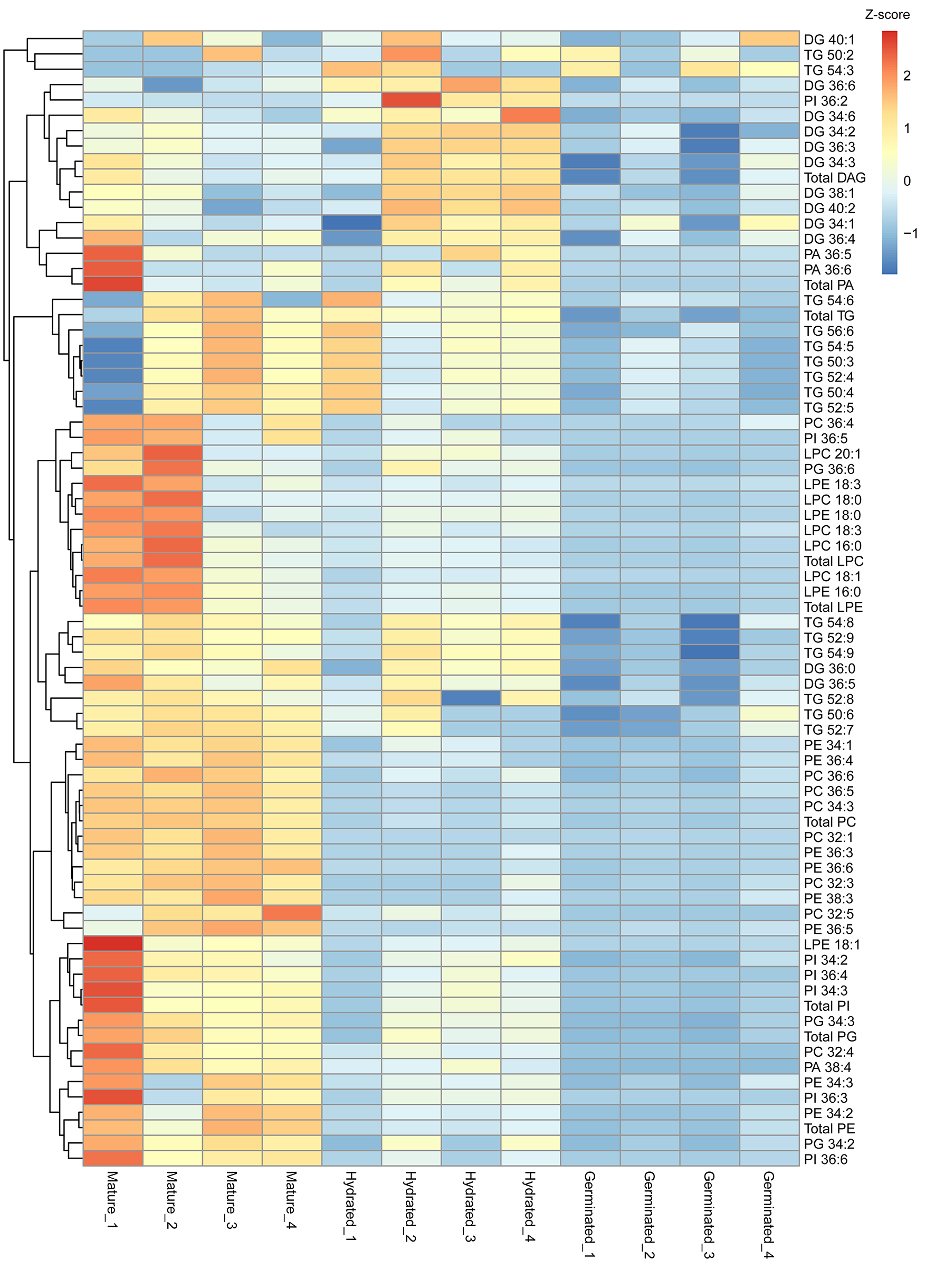

Supplement: Supplementary Figure 1 — Heatmap showing the identified lipid species differences among mature pollen, hydrated and germinated pollen. Heatmap was carried out on raw intensity data converted to z-scores for each metabolite using the “pheatmap” package in R. The scale bar represents the distance between raw intensity and the population mean in units of the standard deviation for a given metabolite. Z-score is negative when the raw intensity is below the mean, positive when above. DG, diacylglycerol; TG, triacylglycerol; PA, phosphatidic acid; PC, phosphatidylcholine; PI, phosphatidylinositol; PG, phosphatidylglycerol; PE, phosphatidylethanolamine; LPC, lysophosphatidylcholine; LPE, lysophosphatidylethanolamine. [file Image_1.TIF]

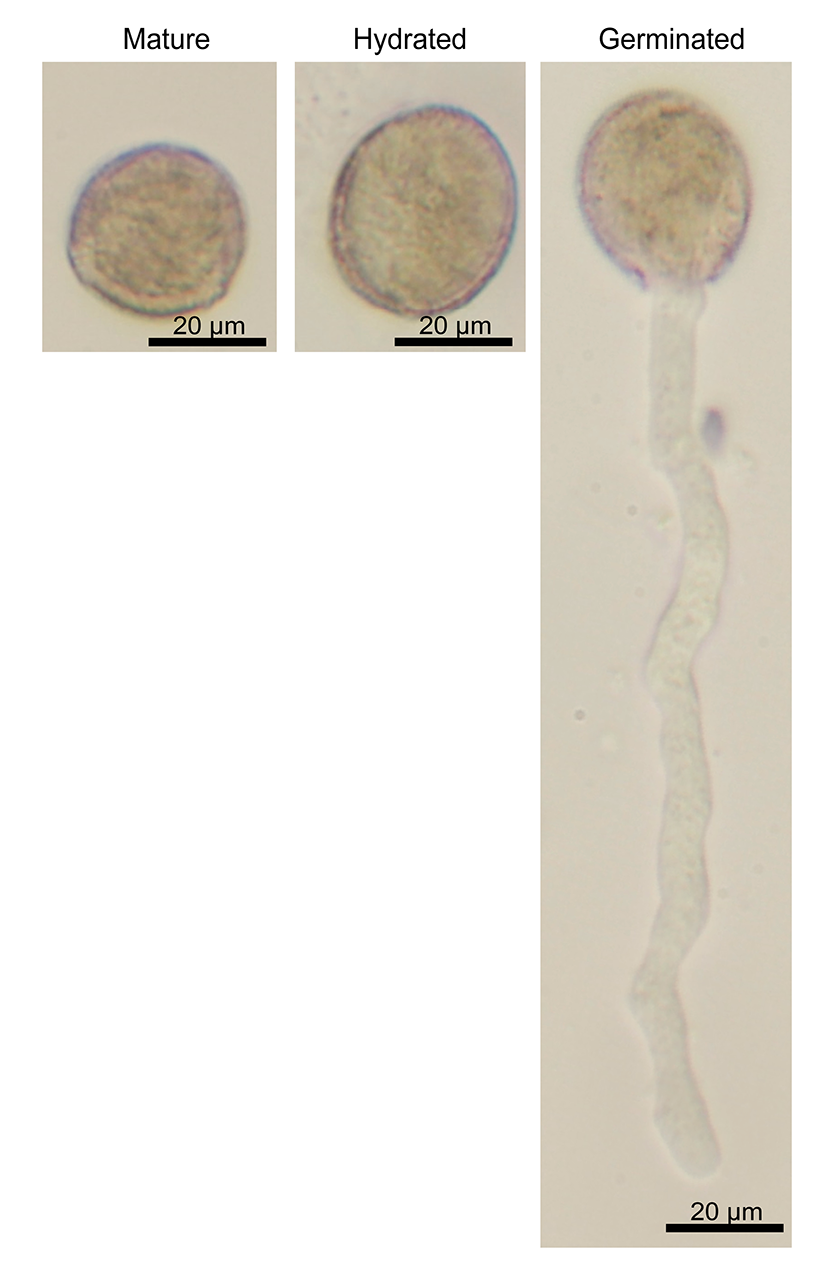

Supplement: Supplementary Figure 2 — Starch staining of mature, hydrated, and germinated pollen. Mature, hydrated, and germinated pollen collected from 50 flowers were stained using iodine solution. No clear starch stains (dark purple) were observed from mature, hydrated, and germinated pollen. [file Image_2.TIF]
